# 

**Published:** 1857-11

**Authors:** 


### CONTENTS FOR NOVEMBER.

Celibacy........................ 245
Various Consumption Cures....... 248
Cold Bathing.................... 251
Wearing Flannel................. 254
How to Arrest a Cold............ 255
Amusing Age Table............... 257
True Temperance................. 258
Scalds and Burns................ 259
Material for Men................ 260
Marriage, Want, and Crime....... 260
The Model Man................... 261
From Laurie Todd................ 262
The Three Glories............... 263
Time Table...................... 264
Agricultural Journals........... 265
Ruin of Young Men............... 246
Webster's Dictionary............ 267
Reviews, Notices, &c............ 268

